# Nasal Morphology in Relation to Vertical Facial Growth Patterns in a Syrian Population

**DOI:** 10.7759/cureus.107170

**Published:** 2026-04-16

**Authors:** Noura Mousa, Abdul-Kareem Hasan

**Affiliations:** 1 Department of Orthodontics, Lattakia University, Lattakia, SYR

**Keywords:** cephalometric study, dentistry, facial growth pattern, nasal morphology, orthodontics

## Abstract

Background

This study aimed to evaluate nasal morphological characteristics and their relationship with vertical facial growth patterns in a Syrian adult population using lateral cephalometric analysis.

Methodology

This retrospective descriptive study included 120 lateral cephalometric radiographs of adult subjects (60 males and 60 females). Subjects were classified into three vertical growth pattern groups according to Björk’s sum (hyperdivergent, normodivergent, and hypodivergent). Linear and angular nasal variables were traced and measured on lateral cephalograms.

Results

Significant sex differences were observed in nasal length, nasal depth, contour of nasal profile, and nasal tip angle. Vertical growth patterns showed a statistically significant association with nasal tip angle, while other nasal variables were not significantly affected.

Conclusions

Nasal morphology differs significantly between sexes and varies according to vertical facial growth patterns. These findings may aid orthodontists in diagnosis and treatment planning aimed at achieving optimal facial aesthetics.

## Introduction

The nose is the most prominent feature of the facial profile and occupies a central position in the face [1-3]. Consequently, it represents a defining characteristic of the midface and plays a critical role in facial aesthetics [1]. It is considered by some clinicians as the keystone of facial esthetics [4].

The main difference between an attractive face and a less attractive one is not determined solely by nasal proportions but rather by the relationship between nasal dimensions and the overall craniofacial complex. Furthermore, the concept of an ideal nose varies among different races, sexes, and ethnic groups. These variations are primarily related to differences in nasal width, protrusion, and the longitudinal axis of the nostrils [2].

Recently, enhancing facial aesthetics has become one of the primary goals of orthodontic treatment [5]. There is an increasing demand for orthodontic therapy to obtain ideal facial proportions, as a result of which the quality of social life is enhanced [6].

The main goal of orthodontic treatment and orthognathic surgical procedures is to achieve a balanced and harmonious face [7], thereby improving patients’ lives through enhanced dentofacial functions and aesthetics [5]. Most patients seek orthodontic treatment primarily for aesthetic reasons, and pleasing soft tissue is considered to be an important factor for a successful treatment [8].

Facial and dental morphological classifications are routinely used in orthodontic practice for diagnosis, treatment planning, and prognosis. Schudy first characterized the interaction between vertical and anteroposterior facial growth as patterns of facial divergence (i.e., hyperdivergent, normodivergent, and hypodivergent), and since then, additional terminology has been used to describe similar morphologic patterns. These classifications are clinically significant due to their association with dental malocclusion and various functional and aesthetic issues [9,10].

Understanding differences in morphology and growth associated with vertical facial divergence patterns is critical for providing appropriate treatment and prognoses for participants [9]. Thus, an orthodontist should have a comprehensive knowledge and understanding of the soft tissue changes that may occur during treatment, and the growth and age-related changes in nasal morphology [11].

A study by Shi et al. investigated the effect of vertical facial growth pattern on the developmental relationship between the nasal bone and maxillary central incisors. The authors reported that nasal bone length was significantly greater in individuals with a hyperdivergent facial pattern compared with those exhibiting hypodivergent and normodivergent patterns [12]. Similarly, Munshi et al. demonstrated that nasal morphological variables are influenced by facial growth pattern and show significant sexual dimorphism [8]. Other studies evaluating the influence of facial growth pattern on nasal morphology have also identified a significant association between nasal length and anterior facial height [13].

Despite numerous studies evaluating nasal morphology, limited data are available regarding its relationship with overall facial growth patterns in the Syrian population. Therefore, this study aimed to assess the relationship between nasal morphological characteristics and vertical facial growth patterns in a sample of the Syrian population.

## Materials and methods

This retrospective descriptive study was conducted using 120 lateral cephalometric radiographs of adult subjects (60 males and 60 females). The study was approved by the Institutional Review Board of Lattakia University (approval number: 4853/2023). The inclusion and exclusion criteria are summarized in Table 1.

**Table 1:** Inclusion and exclusion criteria.

Inclusion criteria	Exclusion criteria
Adults in the post-pubertal stage (cervical vertebral maturation stage 4)	Craniofacial anomalies
High-quality lateral cephalometric radiographs taken in centric occlusion	Systemic diseases
No history of orthodontic treatment or facial surgery	Previous facial trauma
-	Cosmetic surgery
-	Orthodontic treatment involving tooth extractions

The study sample was divided into males and females to assess sex differences. In addition, subjects were classified into three vertical facial patterns based on Björk’s sum, namely, hyperdivergent (Björk sum > 402°), normodivergent (390° < Björk sum < 402°), and hypodivergent group (Björk sum < 390°). All landmarks and linear and angular measurements are described in Table 2.

**Table 2:** Definition of landmarks and measurements used in the study.

Landmarks	Description
N´	Soft‑tissue nasion (the point of greatest concavity in the midline between the forehead and the nose)
Pr	Pronasale (the tip of the nose/nasal tip)
Sn	Subnasale (the deepest point at which the columella merges with the upper lip in the midsagittal plane)
PCm	Posterior columella point (the most posterior point of the lower border of the nose at which it begins to turn inferiorly to merge with the philtrum of the upper lip)
Ls	Labrale superius (the point indicating the mucocutaneous border of the upper lip)
N Lth	Nasal length (the distance between N´ and Pr)
N Dpt	Nasal depth (the perpendicular distance between Pr and the line drawn through N´ to Sn)
CNP	Contour of the nasal profile (the amount (in mm) of soft tissue above the plane connecting N´ to Pr)
NLA	Nasolabial angle (the angle formed by the intersection of the PCm tangent (a tangent drawn from PCm along the lower border of the nose at the approximate middle third) and the PCm-Ls line)
NTP	Nasal tip angle (the angle formed by the axis of the dorsum and PCm tangent)

**Figure 1:**
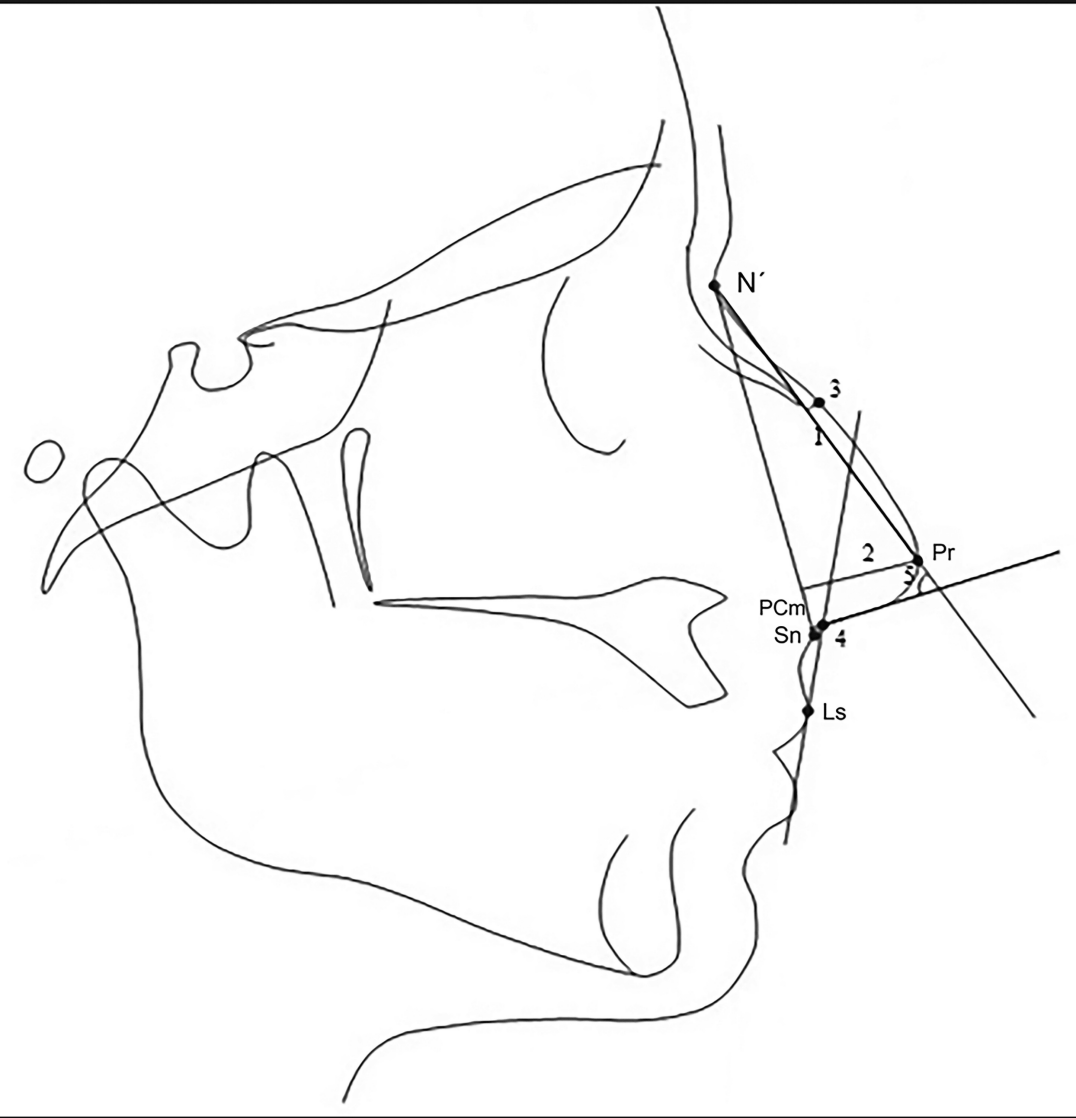
Soft-tissue landmarks and variables used to assess nose on the lateral cephalogram. Nasal length (1). Nasal depth (2). Contour of the nasal profile (3). Nasolabial angle (4). Nasal tip angle (5). N´: soft‑tissue nasion; Pr: pronasale; Sn: subnasale; PCm: posterior columella point; Ls: labrale superius

Cephalometric landmarks and nasal variables were identified and measured, as shown in Figure 1, using Facad software version 3.14 (Ilexis AB, Linköping, Sweden).

To evaluate the reliability of the cephalometric analysis, 10 randomly selected lateral cephalometric radiographs were retraced by the same investigator one month after the initial tracing. The results were compared using the intraclass correlation coefficient analysis.

Statistical analysis

Data were analyzed using SPSS Statistics software for Windows, version 20 (IBM Corp., Armonk, NY, USA). The normal distribution of the data was examined using the Shapiro-Wilk test. Independent-samples t-test and one-way analysis of variance (ANOVA) were used in the case of normality assumptions, and non-parametric Kruskal-Wallis and Mann-Whitney U tests, which are the equivalent of parametric tests, were used in cases that did not provide normal distribution. Statistical significance was set at a p-value <0.05.

## Results

The results of the ICC analysis indicated that all correlation coefficient values fell within the acceptable range, with values ranging from 0.72 to 0.99, which indicates good to excellent reliability, as shown in Table 3.

**Table 3:** Intraclass correlation coefficient results. *: Statistically significant at p < 0.05.

Variable	Intraclass correlation	95% confidence interval		P-value
		Lower bound	Upper bound	
1	0.98	0.92	0.99	<0.001*
2	0.95	0.83	0.98	<0.001*
3	0.85	0.50	0.96	0.001*
4	0.76	0.33	0.93	0.003*
5	0.88	0.60	0.96	<0.001*
6	0.90	0.67	0.97	<0.001*
7	0.92	0.73	0.98	<0.001*
8	0.97	0.92	0.99	<0.001*
9	0.72	0.24	0.92	0.006*

The Shapiro-Wilk test indicated a normal distribution for nasolabial angle and nasal tip angle, while nasal length, nasal depth, and contour of the nasal profile showed non-normal distributions (Table 4).

**Table 4:** Shapiro-Wilk test results for normality. Data represented as length (mm) and degrees (°). *: Statistically significant at p < 0.05. N Lth: nasal length; N Dpt: nasal depth; NLA: nasolabial angle; NTP: nasal tip angle; CNP: contour of the nasal profile; df: degree of freedom

Variable	W	df	P-value
N Lth (mm)	0.98	120	0.04*
N Dpt (mm)	0.97	120	0.01*
NLA (°)	0.99	120	0.19
NTP (°)	0.98	120	0.16
CNP (mm)	0.96	120	<0.001*

The independent samples t-test revealed a statistically significant sex difference in nasal tip angle values, with higher values observed in females (p < 0.05), whereas no significant difference was found for nasolabial angle (Table 5).

**Table 5:** Independent t-test comparing normally distributed variables between males and females. Data represented as degrees (°). *: Statistically significant at p < 0.05. NLA: nasolabial angle; NTP: nasal tip angle; df: degree of freedom

Variable	t	df	P-value	Mean difference
NLA (°)	-1.02	118	0.31	-1.93
NTP (°)	3.17	118	0.001*	4.57

Mann-Whitney U test results demonstrated significant sex differences in nasal length, nasal depth, and contour of the nasal profile (p < 0.05), with higher values observed in males (Table 6).

**Table 6:** Mann-Whitney U test comparing non-normally distributed variables between males and females. Data represented as length (mm). *: Statistically significant at p < 0.05. N Lth: nasal length; N Dpt: nasal depth; CNP: contour of the nasal profile

Variable	U	Z	P-value
N Lth (mm)	1,094.50	-3.71	<0.001*
N Dpt (mm)	1,011.00	-4.18	<0.001*
CNP (mm)	1,042.00	-4.08	<0.001*

One-way ANOVA showed a statistically significant difference among vertical growth pattern groups only for nasal tip angle (p < 0.05), while no significant differences were observed for nasolabial angle (Table 7).

**Table 7:** One-way ANOVA comparing normally distributed variables among vertical growth patterns. Data represented as degrees (°). *: Statistically significant at p < 0.05. NLA: nasolabial angle; NTP: nasal tip angle; ANOVA: analysis of variance

Variable	F	P-value
NLA (°)	0.33	0.72
NTP (°)	3.57	0.03*

The Kruskal-Wallis test revealed no statistically significant differences in nasal length, nasal depth, or contour of the nasal profile among the three vertical growth pattern groups (Table 8).

**Table 8:** Kruskal-Wallis test comparing non-normally distributed variables among vertical growth patterns. Data represented as length (mm). N Lth: nasal length; N Dpt: nasal depth; CNP: contour of the nasal profile; df: degree of freedom

Variable	Chi-square	df	P-value
N Lth (mm)	0.98	2	0.61
N Dpt (mm)	2.80	2	0.25
CNP (mm)	1.10	2	0.58

## Discussion

The present retrospective study was conducted on 120 lateral cephalometric radiographs of adult individuals to evaluate nasal morphology across different vertical facial growth patterns.

Several methods are available for nasal assessment. In addition to lateral cephalometric radiography, standardized photographic analysis, cone-beam computed tomography (CBCT), direct clinical measurements, and nasolabial models can be used. Although the use of 3D data is increasing nowadays to eliminate some of the disadvantages of 2D cephalometric analysis, their widespread use remains limited due to higher radiation exposure compared with lateral cephalograms, as well as the lack of standardized tools for analyzing patient records and CBCT data [11].

In the present study, lateral cephalometric radiographs were used to assess nasal variables because they are routinely obtained for orthodontic diagnosis and treatment planning, involve no additional cost, have no side effects, and provide a practical and reliable method for both clinicians and patients. Moreover, these radiographs can be standardized and have demonstrated acceptable measurement reliability.

The results of this study revealed statistically significant sex differences in nasal tip angle, with higher values observed in females. In contrast, nasal length, nasal depth, and contour of the nasal profile were significantly greater in males. These findings indicate that males tend to have noses that are longer, more prominent, and more convex, whereas females typically exhibit a shorter nose with a more upturned nasal tip.

These results are consistent with those reported by Ocak et al., who found that males exhibited greater linear nasal measurements than females across all evaluated variables [11]. Similar findings were reported by Patel et al., who demonstrated significant sex differences in nasal proportions using lateral cephalometric analysis, with males presenting longer and more prominent noses than females [14]. Additionally, Gulsen et al. reported statistically significant sex-related differences in nasal length, nasal depth, and nasal hump [15].

In contrast, the findings of the present study differ from those of Bhardwaj et al., who reported no statistically significant differences in nasal variables between adult males and females [16]. This discrepancy may be attributed to ethnic differences between the studied populations, as the previous study was conducted on a mixed Indian population, whereas the present study evaluated individuals from the Syrian population.

Regarding vertical facial growth patterns, the results demonstrated a statistically significant difference among the three vertical growth groups in nasal tip angle values. The highest values were observed in the vertical growth pattern group, followed by the normal growth pattern group, while the lowest values were recorded in the horizontal growth pattern group. These findings suggest that individuals with a vertical facial growth pattern tend to exhibit an upturned nose.

This observation is in agreement with the findings of Bhardwaj et al., who reported higher values of both the nasolabial angle and the angle between the lower nose and the Frankfort horizontal plane in adults with vertical growth patterns, resulting in an upturned nasal appearance [16]. Similar results were also reported by Munshi et al., who demonstrated that nasal tip angle values were significantly greater in cases with increased mandibular plane angle [8].

However, the findings of the present study differ from those reported by Rathi et al., who observed that individuals with vertical growth patterns or high-angle cases exhibited a greater tendency toward nasal dorsum convexity compared with those with horizontal growth patterns or low-angle cases [17]. This discrepancy may be explained by differences in sample size and study design.

The present study analyzed 120 lateral cephalometric radiographs, whereas the previous study included only 27 radiographs. Furthermore, the current study incorporated subjects representing all three growth patterns (horizontal, normal, and vertical), while the earlier study was limited to skeletal Class II individuals with only horizontal and vertical growth patterns.

The results of the present study also differ from those reported by Khare et al., who found that both nasal tip angle and nasal upward tip angle were greater in low-angle cases, indicating a more upturned nose in individuals with a horizontal growth pattern. In contrast, the present study demonstrated that an upturned nasal tip was more strongly associated with a vertical facial growth pattern [18]. This discrepancy may be attributed to differences in sample composition, as the current study included individuals with horizontal, normal, and vertical growth patterns, whereas the previous study evaluated only low- and high-angle cases corresponding to horizontal and vertical growth patterns.

Limitations

The retrospective nature of this study limits the generalizability of the findings. The present study used lateral cephalograms for analysis; however, 3D imaging techniques may provide transversal nasal assessments.

## Conclusions

Within the limitations of this study, males demonstrated longer, more prominent, and more convex nasal profiles than females. However, females tended to have shorter, less prominent noses with an upturned nasal tip. Moreover, vertical facial growth patterns were associated with an upturned nasal tip, whereas horizontal growth patterns were associated with a downturned nasal tip.

## References

[1] Naini FB, Naini HB (2011). Facial Aesthetics: Concepts & Clinical Diagnosis. Wiley-Blackwell: Chichester, West Sussex, UK Ames, Iowa.

[2] Jankowska A, Janiszewska-Olszowska J, Grocholewicz K (2021). Nasal morphology and its correlation to craniofacial morphology in lateral cephalometric analysis. Int J Environ Res Public Health.

[3] Jankowska A, Janiszewska-Olszowska J, Jedliński M, Grocholewicz K (2021). Methods of analysis of the nasal profile: a systematic review with meta-analysis. Biomed Res Int.

[4] Sahoo R, Parihar AV, Chaturvedi T, Verma S (2021). Relationship of nasal morphology with different dentoskeletal patterns: a systematic review. J Indian Orthod Soc.

[5] Prasad M, Chaitanya N, Reddy KP, Talapaneni AK, Myla VB, Shetty SK (2014). Evaluation of nasal morphology in predicting vertical and sagittal maxillary skeletal discrepancies'. Eur J Dent.

[6] Jamshed S, Awaisi ZH, Ahmed Z (2023). Lateral cephalometric analysis of nasal morphology in patients visiting Nishtar Institute of Dentistry: lateral cephalometric analysis of nasal morphology. Pak J Health Sci.

[7] Faryal A, Shaikh A (2023). Nasal morphology as an indicator of maxillomandibular skeletal pattern. J Coll Physicians Surg Pak.

[8] Munshi R, Bansal N, Sunda S, Singh G, Chaudhary A, Chuchra A (2022). Does nasal morphology gets affected by growth pattern, skeletal malocclusion and gender dimorphism in an individual in north Indian population: a cephalometric study. Int J Health Sci.

[9] Knigge RP, McNulty KP, Oh H (2021). Geometric morphometric analysis of growth patterns among facial types. Am J Orthod Dentofacial Orthop.

[10] Schudy FF (1964). Vertical growth versus anteroposterior growth as related to function and treatment. Angle Orthodont.

[11] Ocak Y, Cicek O, Ozkalayci N, Erener H (2023). Investigation of the relationship between sagittal skeletal nasal profile morphology and malocclusions: a lateral cephalometric film study. Diagnostics (Basel).

[12] Shi J, Al-Ak'hali MS, Cai D (2023). Effect of the vertical facial pattern on the developmental relationship between the nasal bone and maxillary central incisors. BMC Oral Health.

[13] Nehra K, Sharma V (2009). Nasal morphology as an indicator of vertical maxillary skeletal pattern. J Orthod.

[14] Patel R, Paria P, Mehta F, Parekh H, Kathiriya V (2020). A cross sectional lateral cephalometric study to investigate gender differences for nasal proportions. Int J Creative Res Thoughts.

[15] Gulsen A, Okay C, Aslan BI, Uner O, Yavuzer R (2006). The relationship between craniofacial structures and the nose in Anatolian Turkish adults: a cephalometric evaluation. Am J Orthod Dentofacial Orthop.

[16] Bhardwaj A, Maurya R, Nehra K, Mitra R, Kamat U, Nakra O (2018). Comparative evaluation of various nasal parameters in different malocclusion and growth patterns: a cross-sectional study. J Indian Orthodont Soc.

[17] Rathi S, Gilani R, Kamble R, Vishnani R (2022). Analysis of nasal morphology in skeletal Class I and skeletal Class II malocclusion: an observational study. Cureus.

[18] Khare V, Niwlikar KB (2017). Effect of vertical maxillary skelatal pattern on nasal morphology in high and low angle cases. Int J Oral Health Med Res.

